# Gonadotropin-releasing hormone 2 suppresses food intake in the zebrafish, *Danio rerio*

**DOI:** 10.3389/fendo.2012.00122

**Published:** 2012-10-17

**Authors:** Ryo Nishiguchi, Morio Azuma, Eri Yokobori, Minoru Uchiyama, Kouhei Matsuda

**Affiliations:** ^1^Laboratory of Regulatory Biology, Graduate School of Science and Engineering, University of ToyamaToyama, Japan; ^2^Laboratory of Regulatory Biology, Graduate School of Innovative Life Science, University of ToyamaToyama, Japan

**Keywords:** zebrafish, GnRH2, food intake, ICV administration, Antide, anorexigenic action

## Abstract

Gonadotropin-releasing hormone (GnRH) is an evolutionarily conserved neuropeptide with 10 amino acid residues, of which several structural variants exist. A molecular form known as GnRH2 ([His^5^ Trp^7^ Tyr^8^]GnRH, also known as chicken GnRH II) is widely distributed in vertebrates except for rodents, and has recently been implicated in the regulation of feeding behavior in goldfish. However, the influence of GnRH2 on feeding behavior in other fish has not yet been studied. In the present study, therefore, we investigated the role of GnRH2 in the regulation of feeding behavior in a zebrafish model, and examined its involvement in food intake after intracerebroventricular (ICV) administration. ICV injection of GnRH2 at 0.1 and 1 pmol/g body weight (BW) induced a marked decrease of food consumption in a dose-dependent manner during 30 min after feeding. Cumulative food intake was significantly decreased by ICV injection of GnRH2 at 1 pmol/g BW during the 30-min post-treatment observation period. The anorexigenic action of GnRH2 was completely blocked by treatment with the GnRH type I receptor antagonist Antide at 25 pmol/g BW. We also examined the effect of feeding condition on the expression level of the GnRH2 transcript in the hypothalamus. Levels of GnRH2 mRNA obtained from fish that had been provided excess food for 7 days were higher than those in fish that had been fed normally. These results suggest that, in zebrafish, GnRH2 acts as an anorexigenic factor, as is the case in goldfish.

## INTRODUCTION

Gonadotropin-releasing hormone (GnRH) is an evolutionarily conserved decaneuropeptide that plays a crucial role in the regulation of reproduction in vertebrates (Sherwood et al., 1993; Fernald and White, 1999, Millar et al., 2004). The demonstration of GnRH structural variants in vertebrates, and even in invertebrates, has now resulted in the identification of 29 molecules (Guilgur et al., 2006; Kah et al., 2007; Roch et al., 2011). In vertebrates, these peptides are distributed in a wide range of tissues, and have diverse functions as hypophysiotropic hormones, paracrine or autocrine mediators and neuromodulators/neurotransmitters in the central and peripheral nervous systems and tissues (Gore, 2002; Millar, 2005; Kim et al., 2007; Millar et al., 2007). GnRH with substitutions at the N-terminal fifth, seventh, and eighth positions by histidine, tryptophan, and tyrosine residues, respectively, was originally purified and characterized as a second type of GnRH from chicken brain, and was named chicken GnRH II (now called GnRH2; Miyamoto et al., 1984). Subsequently, it has been found that GnRH2 is present throughout the vertebrates from cartilaginous fish to humans, but not in rodents (Conlon et al., 1993; Sherwood et al., 1993; White et al., 1998; Millar, 2003). GnRH2 has been implicated in the central regulation of reproductive behavior as well as neuroendocrine control of the gonads (Maney et al., 1997; Volkoff and Peter, 1999; Schiml and Rissman, 2000; White et al., 2002; Temple et al., 2003; Lethimonier et al., 2004; Hofmann, 2006). Recently, it has been reported that in an insectivore, the musk shrew, GnRH2 also influences feeding behavior, and that intracerebroventricular (ICV) administration of GnRH2 induces a marked decrease in food consumption, suggesting that GnRH2 controls reproduction and energy balance (Temple et al., 2003; Kauffman, 2004; Kauffman and Rissman, 2004a,b; Kauffman et al., 2005b, 2006). However, the involvement of GnRH in the regulation of feeding behavior had not been studied in other animal models.

Previous studies have indicated that ICV injection of GnRH2 also induces an anorexigenic effect in a goldfish model (Hoskins et al., 2008; Matsuda et al., 2008; Kang et al., 2011). In addition to goldfish, the zebrafish has now been widely used as an excellent animal model to investigate the effects of neuropeptides on feeding behavior (Yokobori et al., 2011, 2012; Matsuda et al., 2012). As in rodents and goldfish, it has been found that, in zebrafish, neuropeptide Y (NPY) and orexin A stimulate food intake (Yokobori et al., 2011, 2012; Matsuda et al., 2012). However, the exact role of GnRH2 is unclear, and there is no information about the effect of GnRH2 on feeding behavior in this species.

Therefore, the aim of the present study was to investigate the effect of GnRH2 on food intake in the zebrafish model, and the effect of ICV injection of Antide, a GnRH type I receptor antagonist, on the action of ICV-administered GnRH2. We also examined the effect of feeding condition on the expression level of the GnRH2 transcript in the hypothalamus.

## MATERIALS AND METHODS

### ANIMALS

Adult zebrafish (*Danio rerio*, 0.5–1.0 g body weight, BW) of both sexes were obtained commercially, and kept for 2 weeks under controlled light/dark conditions (12 h light/12 h dark) in a water-temperature-regulated fish tank (20–24°C) before use in experiments, since prevention of gonadal development. The fish were fed a commercially available granule diet (containing 32% protein, 4% dietary fat, 3% dietary fiber, 9% mineral, 8% water, and 44% other components; Hikari MariGold, Kyorin, Kobe, Japan) every day at noon. For 1 week before the experiments each fish was kept in a small experimental tank (24 cm in diameter) with 3.5 l of tap water. All animal experiments were conducted in accordance with the University of Toyama guidelines and the Declaration of Helsinki for the care and use of animals. Every effort was made to minimize the number of animals used and their suffering.

### CHEMICALS

The zebrafish possesses two molecular forms of GnRH: GnRH2 (pGlu-His-Trp-Ser-His-Gly-Trp-Tyr-Pro-Gly-NH_2_), and GnRH3 (pGlu-His-Trp-Ser-Tyr-Gly-Trp-Leu-Pro-Gly-NH_2_; Powell et al., 1996; Steven et al., 2003). Therefore, in order to examine the effect of ICV injection of GnRH2 on food intake, GnRH2 was purchased from Bachem AG (Bubendorf, Switzerland) and used. Zebrafish possesses four kinds of GnRH receptors (GnRH R1–R4), GnRH R1 and R3 being of the GnRH type I receptor type whereas GnRH R2 and R4 are GnRH type III receptors (Tello et al., 2008). In the present study, we used the GnRH type I receptor antagonist, Antide (acetyl-D-Ala(2-naphthyl)-D-Phe(4-Cl)-D-Ala(3-pyridyl)-Ser-Lys(Nε-nicotinoyl)-D-Lys(Nε-nicotinoyl)-Leu-Lys(Nε-isopr-opyl)-Pro-D-Ala-NH_2_), obtained from Sigma-Aldrich Co. (St. Louis, MO, USA). Antide was dissolved in 0.1% acetic acid and diluted with 0.6% NaCl and 0.02% Na_2_CO_3_ solution (saline) before use.

### EFFECT OF ICV ADMINISTRATION OF GnRH2 ON FOOD INTAKE

**FIGURE 1:**
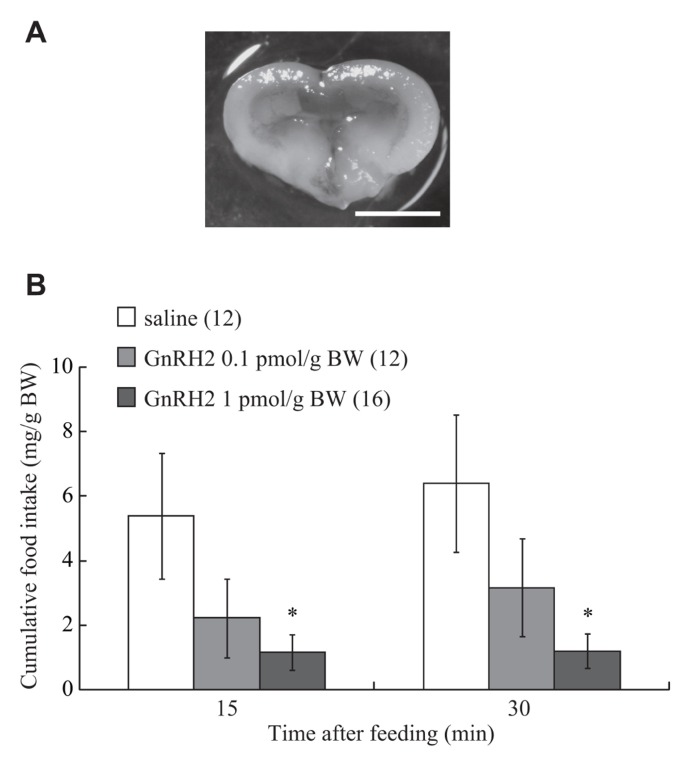
**(A)** A photograph showing ICV administered-solution containing 0.05% Evans Blue into the brain. Scale bar is 1 mm. **(B)** Effect of ICV administration of GnRH2 on food intake in the zebrafish. Each column and bar represents the mean and SEM, respectively, and the numbers in parentheses in the panels indicate the number of fish in each group. Significances of differences at each time point were evaluated by one-way ANOVA with the Bonferroni’s method in comparison with the vehicle-injected group (**P < 0.01).

Details of the methods used for evaluating feeding behavior in zebrafish have been reported elsewhere (Yokobori et al., 2011, 2012). Each fish was normally fed before the experiments began at noon, and placed in a wet sponge under anesthesia with MS-222 (3-aminobenzoic acid ethyl ester; Sigma-Aldrich). A small part of the parietal bone was carefully removed using a surgical blade (No. 19, Futaba, Tokyo, Japan), and then 0.5 μl/g BW of GnRH2 at doses of 0.1 and 1 pmol/g BW was injected into the third ventricle of the brain using a small Hamilton syringe. The gap in the bone was then filled with a surgical agent (Aron Alpha, Sankyo, Japan). The accuracy of the injection site was confirmed after the experiment by examining whether Evans blue dye, injected at the same time, was present in the ventricle (**Figure 1A**). Control fish in each experiment were injected with the same volume of vehicle (less than 0.01% acetic acid diluted with saline) in the same way as for the experimental group. Each fish that had received an injection was individually placed in a small experimental tank (24 cm in diameter) containing 3.5 l of tap water, and supplied with food equivalent to 3% of its BW. Food intake was measured by directly observing and recording the number of diet pellets eaten by individual fish over 15 and 30 min of commencement of feeding.

### EFFECT OF ICV INJECTION OF ANTIDE ON GnRH2-INDUCED ANOREXIGENIC ACTION

Because pilot experiments had shown that ICV injection of cGnRH2 at a dose of 1 pmol/g BW induced a marked decrease of food intake, Antide at 25 pmol/g BW, a dose previously determined to be sufficient to suppress the action of GnRH2 in goldfish (Matsuda et al., 2008), was delivered by ICV injection in addition to GnRH2 at 1 pmol/g BW. Control fish were injected with the same volume of vehicle (less than 0.01% acetic acid diluted with saline) in the same way as for the experimental group. Food intake was then measured over the first 15 and 30 min of commencement of feeding, as described above.

### EFFECT OF FEEDING CONDITION ON GnRH2 mRNA EXPRESSION IN THE HYPOTHALAMUS

Every day for 7 days, fish were supplied an excessive amount of food corresponding to 9% of their BW. Other fish were fed an amount of food corresponding to 3% of their BW for the same period. On day 7, the fish were anesthetized with MS-222 and decapitated. Because the zebrafish brain is very small, each brain was dissected out, and the hypothalamus was collected, weighed, and immersed immediately in liquid nitrogen, before being stored at -80°C until use. Total RNA was extracted from each part of the brain with Isogen (a solution containing phenol and guanidinium isothiocyanate; Nippon Gene, Tokyo, Japan). For amplification and quantitation of the cDNA fragments encoding GnRH2 and β-actin, the one-step reverse transcription polymerase chain reaction (RT-PCR) method (SYBR Green RT-PCR Reagents Kit, Applied Biosystems, Foster City, CA, USA) was used. Reactions (including 5 μM primers, 2× SYBR Green PCR master mix, 6.25 U MultiScribe reverse transcriptase, 10 U RNase inhibitor, RNA template, and water) were set up in a 96-well reaction plate and placed in a sequence detection system for cycling (TP 800, Takara, Tokyo, Japan). Reverse transcription was carried out at 48°C for 30 min and the resulting cDNA was subsequently amplified using 40 cycles of 95°C for 15 s followed by 60°C for 60 s. The PCR products from each cycle were monitored using SYBR Green I fluorescent dye (Applied Biosystems). Gene-specific primers for amplification of the GnRH2 cDNA fragment were based on the nucleotide sequence of zebrafish GnRH2 (GenBank ID, BC162945.1, NM_181439; Ensembl ID, ENSDARG00000044754). PCR with the sense primer (5′-CAA AAT ATT AGA CTG AAG TGA TGG T-3′) and the antisense primer (5′-GGT CTA TCT CTC TCT TTC CTC CA-3′) yielded a 86-bp product encoding zebrafish GnRH2 cDNA. Zebrafish β-actin-specific primers were used as the internal control for PCR amplification (GenBank accession number, NM_181601; Ensembl ID, ENSDART00000055194). Using these primers (sense primer, 5′-GTG ATG GAC TCT GGT GAT GGT GT-3′; antisense primer, 5′-TGA AGC TGT AGC CTC TCT CGG TC-3′), a 148-bp product corresponding to a region in the central part of the β-actin cDNA sequence was obtained. The expression levels of GnRH2 mRNA were calculated quantitatively as a ratio relative to the expression of β-actin mRNA.

### DATA ANALYSIS

All the results are expressed as mean ± SEM. Statistical analysis was performed by one- and two-way ANOVA with Bonferroni’s method or Student’s *t*-test. Statistical significance was determined at the 5% level.

## RESULTS

### EFFECT OF ICV ADMINISTRATION OF GnRH2 ON FOOD INTAKE

Intracerebroventricular injection of GnRH2 (at 0.1 and 1 pmol/g BW) inhibited food intake over a 30-min feeding period. A significant reduction in cumulative food consumption was observed at a dose of 1 pmol/g BW at both 15 and 30 min after commencement of feeding (**Figure 1B**). The *df*, *F*, and *P* values between treatments with saline and GnRH2 were: 2, 3.08, and 0.06 at 15 min; 2, 3.51, and 0.04 at 30 min.

### EFFECT OF ICV INJECTION OF ANTIDE ON ANOREXIGENIC ACTION OF GnRH2

**FIGURE 2:**
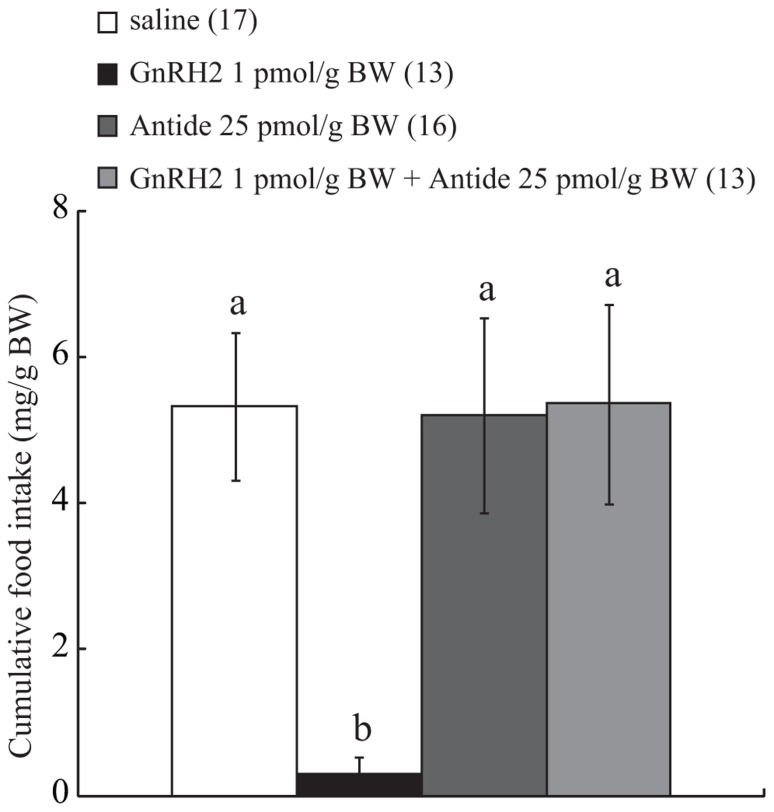
**Effect of ICV administration of Antide on the anorexigenic action of GnRH2**. The results are expressed as a percentage of the control, i.e., vehicle-injected, fish. Each column and bar represents the mean and SEM, respectively, and the numbers in parentheses in the panels indicate the number of fish in each group. Significance of differences among experimental groups was evaluated by two-way ANOVA with the Bonferroni’s method in comparison with the vehicle-injected group (**P < 0.01).

Intracerebroventricular administration of GnRH2 alone at 1 pmol/g BW suppressed food intake over a 30-min feeding period, and ICV-injected Antide alone at 25 pmol/g BW did not affect food intake. On the other hand, the same dose of Antide completely abolished the anorexigenic action of ICV-injected GnRH2 at a dose of 1 pmol/g BW, and the efficacy of the antagonist was shown to be significant by two-way ANOVA with Bonferroni’s method (*df*, *F*, and *P* values, 1, 4.39, and 0.04, respectively; **Figure 2**).

### EFFECT OF FEEDING CONDITION ON GnRH2 mRNA EXPRESSION IN THE HYPOTHALAMUS

**FIGURE 3:**
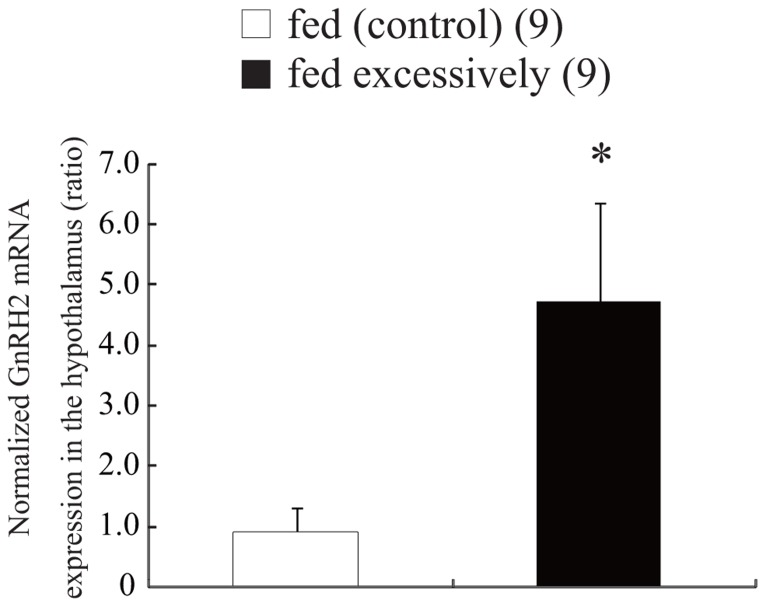
**Effect of feeding condition on the level of expression of GnRH2 mRNA in the hypothalamus**. The results are expressed as the mean ± SEM, and the number of fish per group is indicated in parentheses. Statistical significance was evaluated by Student’s *t*-test (**P < 0.01).

**Figure 3** shows the expression levels of GnRH2 mRNA in the hypothalamus of zebrafish supplied an excessive amount of food corresponding to 9% of their BW, and normal amount of food corresponding to 3% of their BW. Expression of GnRH2 mRNA was estimated quantitatively as a ratio relative to the expression of β-actin mRNA. In the hypothalamus, excessive feeding for 7 days induced a significant increase (approximately four times higher) in the level of GnRH2 mRNA compared with that in fish that had been fed normally (*t* and *P* values, 2.82 and 0.012, respectively; **Figure 3**).

## DISCUSSION

We have developed methods for administering ICV test substances and for measuring food consumption in a small fish, the zebrafish, and our previous studies have demonstrated that, in this species, NPY and orexin A act as orexigenic neuropeptides (Yokobori et al., 2011, 2012). In the present study, we investigated the effect of central administration of GnRH2 on food intake, and demonstrated for the first time that GnRH2 strongly suppresses food consumption in the zebrafish. In matured female musk shrew, an insectivore, ICV injection of GnRH2 inhibits food intake, and feeding status influences the levels of both GnRH2 mRNA expression and immunoassayable GnRH2 in the brain, which are decreased by food restriction (Kauffman and Rissman, 2004a; Kauffman et al., 2006). The present study indicates the anorexigenic action of GnRH2 in the zebrafish, as is the case in female musk shrew and goldfish (Hoskins et al., 2008; Matsuda et al., 2008; Kang et al., 2011), indicating that it may be involved generally in the regulation of feeding behavior in vertebrates. GnRH3 is widely distributed in several regions of the zebrafish brain, including the olfactory bulb, the area of the terminal nerve, and telencephalon, and GnRH3 is implicated in pituitary control (Torgersen et al., 2002; Steven et al., 2003; Palevitch et al., 2007). On the other hand, GnRH2-expressing neurons are localized mainly in the midbrain tegmentum (Palevitch et al., 2007). However, the exact role of GnRH2 in the zebrafish has been unclear. Because GnRH2 is implicated in the regulation of reproductive behavior and energy balance in the female musk shrew, sparrow, and goldfish (Maney et al., 1997; Temple et al., 2003; Kauffman, 2004; Kauffman and Rissman, 2004a; Kauffman et al., 2005a,b; Hofmann, 2006; Hoskins et al., 2008; Matsuda et al., 2008; Kang et al., 2011), it is likely that, in zebrafish, GnRH2 is involved in both feeding control and reproductive behavior. GnRH systems have been well studied in teleost fish (Lethimonier et al., 2004). The zebrafish possesses two GnRHs – GnRH2 and GnRH3 – which are encoded by two distinct genes (Steven et al., 2003), and four GnRH receptors, GnRH R1–R4 – which are members of the G protein-coupled receptor family (Tello et al., 2008). There are marked differences in structure between these two groups of GnRHRs. GnRH R1 and R3 are evolutionarily derived from the common ancestor of the GnRH type I receptor, and GnRH R2 and R4 belong to the GnRH type III receptor group (Tello et al., 2008). In the present study, ICV administration of GnRH2 at 1 pmol/g BW induced a significant decrease of food intake, and this effect was completely blocked by treatment with Antide. Antide is the GnRH type I receptor antagonist (Kauffman et al., 2005b; Matsuda et al., 2008). These results suggest that the anorexigenic action of GnRH2 is mediated by the Antide-sensitive receptor system, perhaps involving GnRH R1 and/or R3. The knowledge about the expression and the distribution of GnRH receptors in the zebrafish brain should help us to speculate on the mechanisms underlying GnRH2-induced anorexigenic effect. However, there has been no information about distribution of GnRH receptors in the hypothalamus. It is unclear which receptor type mediates the anorexigenic action of GnRH2 in zebrafish.

In mammals, several neuropeptides including CRH, galanin-like peptide, LHRH (GnRH1), kisspeptin, α-MSH, NPY, orexin, and 26RFa are implicated in the regulation of nutrition and reproduction, suggesting that feeding and reproductive functions are closely linked (Catzeflis et al., 1993; Iqubal et al., 2001; Chartrel et al., 2003; Kauffman et al., 2005a; Crown et al., 2006; Martynska et al., 2006; Navarro et al., 2006; Maeda et al., 2007). Orexin, which has crucial role in the sleep–wakefulness cycle and appetite control, affects GnRH1 release directly or via the NPY-, CRH-, and β-endorphin-signaling pathways (Li et al., 1999; Tamura et al., 1999; Irahara et al., 2001; Yang et al., 2005; Iwasa et al., 2007). In goldfish, ICV administration of orexin A suppresses spawning behavior, and ICV administration of GnRH2 reduces the level of orexin precursor mRNA in the brain (Hoskins et al., 2008). These data suggest that, in goldfish, GnRH2 and orexin have opposite roles in appetite and satiety regulation. Our previous studies have revealed that mRNA expression levels for NPY and orexin in the hypothalamus obtained from zebrafish fasted for 7 days are higher than those in zebrafish that had been fed normally, suggesting NPY and orexin in the hypothalamus act as orexigenic factors in this species. In the present study of zebrafish, we focused on the GnRH2 neuronal system, which has been implicated in the regulation of food intake in the goldfish (Matsuda et al., 2008), and demonstrated for the first time that the expression of GnRH2 mRNA in the hypothalamus is affected by feeding status. The results support the idea that GnRH2 in the hypothalamus acts as an anorexigenic neuropeptide in this species. Further investigations to clarify the regulatory mechanism of food intake by GnRH2 and other neuropeptides and factors are warranted.

In conclusion, the present study has demonstrated for the first time that ICV administration of GnRH2 suppresses food intake in the zebrafish. These results suggest that GnRH2 induces behavioral changes, and in particular acts as an anorexigenic factor in this species. The present findings also indicate that evolutionary pressure has acted to preserve the function of GnRH2 as a feeding regulator across the vertebrates.

## Conflict of Interest Statement

The authors declare that the research was conducted in the absence of any commercial or financial relationships that could be construed as a potential conflict of interest.
